# In Vivo Evaluation of Wound Healing Efficacy of Gel-Based Dressings Loaded with Pycnogenol™ and *Ceratothoa oestroides* Extracts

**DOI:** 10.3390/gels10040233

**Published:** 2024-03-28

**Authors:** Andreas Vitsos, Dimitra Ieronymaki, Maria Kostaki, Chara Almpani, Christina Barda, Stefanos Kikionis, Ioannis Sfiniadakis, Paraskevas Dallas, Michail Christou Rallis

**Affiliations:** 1 Section of Pharmaceutical Technology, Department of Pharmacy, National and Kapodistrian University of Athens, Panepistimiopolis Zografou, 15784 Athens, Greece; avitsos@yahoo.gr (A.V.); dimitraier93@gmail.com (D.I.); marie.kstk@gmail.com (M.K.); ch.almpani@gmail.com (C.A.); dallas@pharm.uoa.gr (P.D.); 2 Section of Pharmacognosy and Chemistry of Natural Products, Department of Pharmacy, National and Kapodistrian University of Athens, Panepistimiopolis Zografou, 15784 Athens, Greeceskikionis@pharm.uoa.gr (S.K.); 3 Athens Naval Hospital, Pathologoanatomic Laboratory, 11521 Athens, Greece; jsfiniadakis@yahoo.gr

**Keywords:** *Ceratothoa oestroides*, Pycnogenol™, composite wound dressings, wound healing, *Pinus pinaster*, gel dressings

## Abstract

*Ceratothoa oestroides* and French maritime pine bark (Pycnogenol™) extracts are considered promising therapeutic agents in wound healing. This study explores the healing efficacy of composite dressings containing these extracts, aiming to enhance their stability and effectiveness, utilizing a low-temperature vacuum method for producing Sodium Alginate—Maltodextrin gel dressings. Surgical wounds were inflicted on SKH-hr2 hairless mice. Dressings were loaded with Pycnogenol™ and/or *C. oestroides* extracts and assessed for their efficacy. Wound healing was primarily evaluated by clinical and histopathological evaluation and secondarily by Antera 3D camera and biophysical measurements. Dressings were stable and did not compromise the therapeutic properties of *C. oestroides* extract. All interventions were compared to the *C. oestroides* ointment as a reference product. Most of the wounds treated with the reference formulation and the *C. oestrodes* dressing had already closed by the 15th day, with histological scores of 7 and 6.5, respectively. In contrast, wounds treated with Pycnogenol™, either alone or in combination with *C. oestroides,* did not close by the end of the experiment (16th day), with histological scores reaching 15 in both cases. Furthermore, treatment with 5% Pycnogenol™ dressing appeared to induce skin thickening and increase body temperature. The study underscores the wound healing potential of *C. oestroides* extracts and highlights the need for further research to optimize Pycnogenol™ dosing in topical applications.

## 1. Introduction

Wound repair is a multifaceted and dynamic process that involves a plethora of phenomena. Typically, the repair process progresses through four distinct yet overlapping stages. It begins with hemostasis, which is followed by inflammation, progresses with angiogenesis, extracellular matrix deposition, granulation tissue formation, and epithelialization during the proliferative phase, and concludes with connective tissue deposition during the tissue remodeling/maturation phase [1].

In recent decades, numerous research efforts have focused on the discovery of wound healing drugs [2]. Several natural products are regarded as optimal treatments for a broad spectrum of illnesses. Bioactive extracts derived from nature, known for their diverse biological activities, are considered promising for use in medical, pharmaceutical, and biotechnological fields, especially in promoting wound healing and skin regeneration [3,4]. 

Pycnogenol™ is recognized for its potent anti-inflammatory effects, which stem from its ability to block the NF-kB and AP-1 pathways involved in inflammation. By interfering with these pathways, it leads to a decrease in the levels of inflammatory cytokines such as TNF-α, IL-6, and IL-1β, whilst also enhancing healing processes [5]. Kyriazi et al. [6] have shown through in vivo studies that the application of Pycnogenol™ serves as a blockade against the carcinogenic and aging effects on the skin triggered by UV exposure. With its combination of anti-inflammatory, antioxidative, and tissue regeneration capabilities, Pycnogenol™ may be considered an effective agent for therapeutic applications in wound healing [7,8]. Moreover, there are already published results confirming its therapeutic efficacy [8,9,10].

*Ceratothoa oestroides*, an isopod parasite found in the oral cavity of large fish, is currently identified as one of the most significant threats to aquaculture. The olive oil extract of *C. oestroides,* formulated as an ointment, has been studied over the last decade by our laboratories, focusing primarily on its healing properties but also exploring its anti-inflammatory, anti-psoriatic, and anti-atopic dermatitis effects, yielding highly significant outcomes both in preclinical and clinical trials [11,12,13,14,15,16,17].

Alginate gel dressings have become prominent biomaterials with versatile applications due to their unique properties [18]. Derived from kelp, Sargassum algae, and certain bacterial strains, alginates are linear anionic polysaccharides that have undergone extensive research since their discovery in the late nineteenth century [18]. Notably, alginates are non-toxic, readily available from natural sources, and possess biocompatible and biodegradable characteristics within the human body [19]. These favorable attributes have propelled alginates into significant roles within the food industry and as essential biomaterials in pharmaceutical and biomedical fields [19]. Alginate-based hydrogels have garnered attention for their efficacy in wound dressing, tissue engineering, and drug delivery applications [19]. Their ability to form gels in the presence of divalent cations such as calcium ions enables them to create a moist environment conducive to wound healing. They also provide mechanical support and facilitate the controlled release of therapeutic agents [19]. As a result, alginate gel dressings are a promising option in the field of wound care and regenerative medicine, offering both versatility and effectiveness in various clinical settings [18,19].

Pycnogenol and *C. oestroides* extracts have shown their effectiveness in promoting wound healing when formulated as ointments [8,9,10,11,12,13,14,15,16,17]. However, the pharmaceutical form of ointment presents notable drawbacks concerning wound healing. Accurate dosing is challenging, and ointments may be displaced from the affected area due to exudation or absorption into dressings. This study aimed to enhance the pharmaceutical form of the extracts while maintaining their effectiveness, as well as to investigate their combined administration in topically applied formulations. The doses were established by referring to previous relevant studies [10,11], with a specific dosage of 5% for Pycnogenol™ and 10% for the extract of *C. oestroides*. The gel dressings were chosen based on their ability to continuously release the active ingredients that were included, as well as due to their simple preparation.

The study aimed to develop and assess improved topical formulations containing Pycnogenol™ and *C. oestroides* extracts for wound healing, addressing the limitations observed with ointment forms. Specifically, it sought to ensure the effective delivery of these extracts by incorporating them into gel dressings, which offer advantages in terms of accurate dosing and stability at the wound site. By transitioning from an ointment to a gel dressing formulation, the study aimed to maintain the therapeutic benefits of the extracts while overcoming the challenges of ointment use, such as displacement due to wound exudation or dressing absorption. Additionally, the study intended to explore the effects of combining these two extracts in a single formulation, assessing their potential synergistic effects on wound healing.

## 2. Results and Discussion

### 2.1. Dressings Characterization

**Figure 1:**
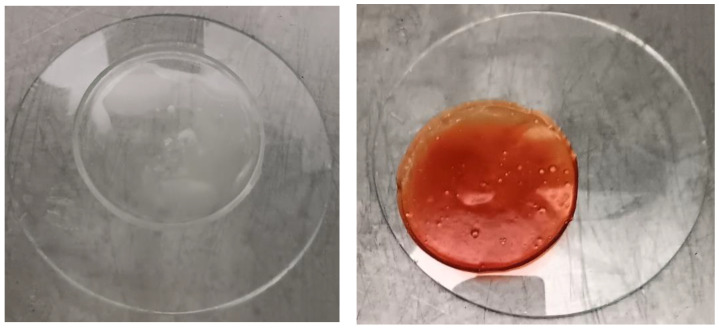
Photographic Capture of Gel Dressings. On the right, a dressing devoid of any active ingredients is highlighted, while on the left, a dressing containing both *C. oestroides* and Pycnogenol™ is depicted.

The produced dressings (Figure 1) exhibited elasticity, malleability, and durability.

**Figure 2:**
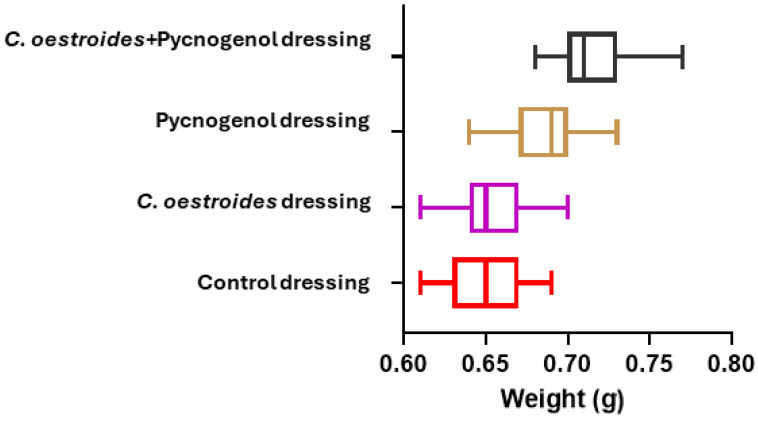
Weight variation of different dressings. Weight measurements are presented in grams (g). The combined dressings (in black) exhibited a higher mean and median weight compared to all other dressings, followed by the Pycnogenol™ dressings (in brown). The dressings containing *C. oestroides* (magenta) and the control dressings (red) demonstrated approximately equal weights.

The manufacturing method yielded patches with notably low weight variation across most of the samples (±3%), which are considered generally acceptable by the European pharmacopeia for the production of solid unit dose pharmaceutical forms (Figure 2).

**Figure 3:**
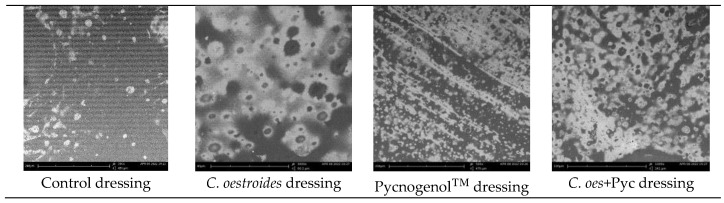
SEM images of the different dressings.

The Scanning Electron Microscope (SEM) images (Figure 3) revealed the smooth surface of the dressings, similar to observations made in other cases of alginate dressings [20], as well as a complete and uniform dispersion of water-soluble components. However, the dispersion of the oily phase appeared to be less uniform across all dressings. It is noted that these observations were not visible to the naked eye. Although further efforts must be made to improve the method, the dispersion of the active ingredients was deemed satisfactory for the needs of the current experiment.

### 2.2. Weight and Temperature Measurements

Regarding the weight of the experimental animals, no statistically significant differences were observed between their initial (mean 30.34 ± 3.07 g) and final weights (mean 31.79 ± 3.31 g) or among the different intervention treatments.

**Figure 4:**
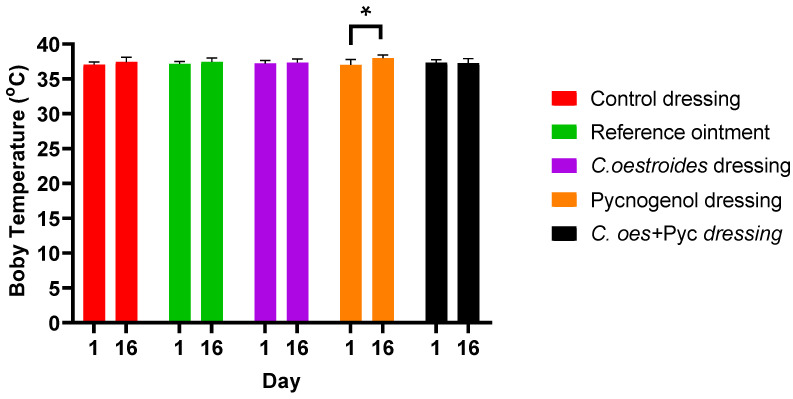
Measurements of the experimental animals’ temperature at the beginning and end of the experiment were maintained at the same levels, apart from Pycnogenol™, which showed a slight increase. * Statistical significance between starting and ending measurements was observed for Pycnogenol™ groups (*p* < 0.05).

Similarly, for temperature, in most cases, no statistically significant differences were noticed between the initial and final measurements (Figure 4). However, the treatment that received the dressing containing only Pycnogenol™ showed a slightly higher temperature at the end of the experiment compared to the initial temperature (*p* < 0.05).

### 2.3. Transepidermal Water Loss

**Figure 5:**
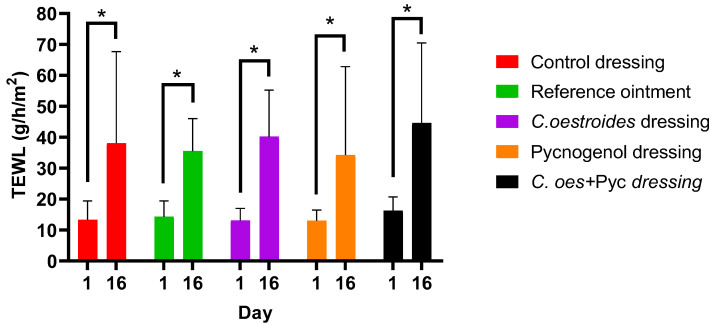
Measurements of the transepidermal water loss (TEWL) before wound infliction and at the end of the study. * Statistical significance between starting and ending measurements was observed in all groups (*p* < 0.05).

In the measurements of transepidermal water loss, a significant difference was observed between the initial measurement and that on the 16th day after the wound induction in all interventions. However, no statistically significant differences were presented between the various interventions (Figure 5).

### 2.4. Hydration

**Figure 6:**
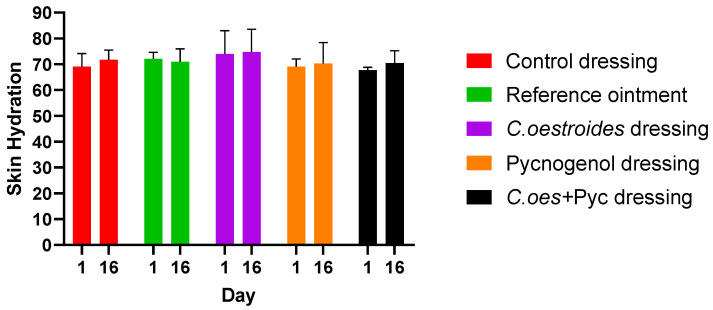
Measurements of the hydration before wound infliction and at the end of the study. No statistically significant differences were observed between the different groups or starting and ending points (*p* > 0.1).

In the hydration measurements, no statistically significant differences were observed between the various interventions (Figure 6). It is worth mentioning that this measurement reflects the stratum corneum water content. On the 16th day, oedema may especially contribute to the presented outcome, as the hydration levels of days 1 and 16 are the same.

### 2.5. Skin Thickness

**Figure 7:**
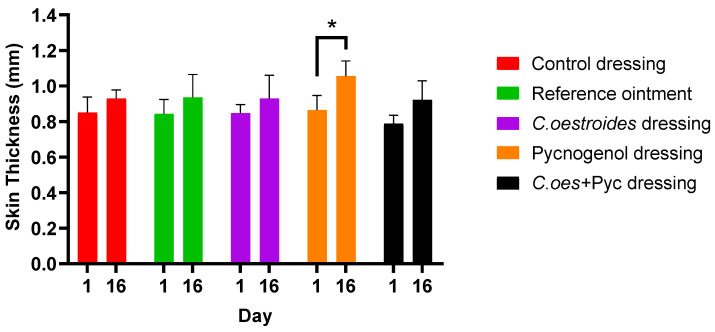
Skin thickness measurements at the start and conclusion of the experiment. * Statistical significance (*p* = 0.0011) reported between starting and ending measurements regarding Pycnogenol™ dressing treatment.

Like the temperature measurements, the skin only became thicker (*p* = 0.0011) in the case of local treatment with the dressing containing Pycnogenol™. No other statistically significant difference was observed (Figure 7).

### 2.6. Wound Area, Volume, and Debt

**Figure 8:**
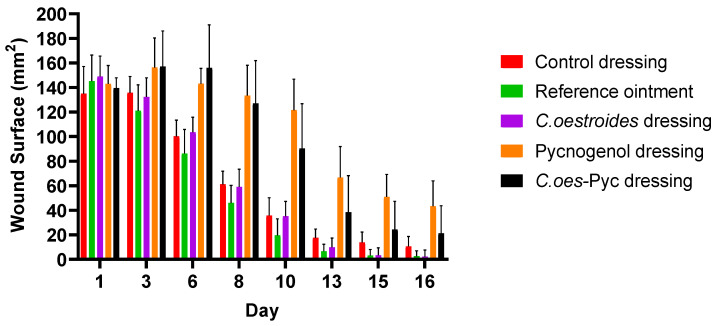
Wound surface area measurements throughout the experiment. Significant differences were observed as follows: by the 13th day, between the reference ointment treatment and the control dressing and the Pycnogenol™ dressings (*p* < 0.01); by the 15th day, the reference ointment treatment, compared to *C. oestroides* dressing (*p* < 0.02). Pycnogenol™ dressings, showed a statistically significant (worsening) compared to the control; between the reference ointment and *C. oestroides* dressing from the 6th day until the final day (*p* < 0.001).

**Figure 9:**
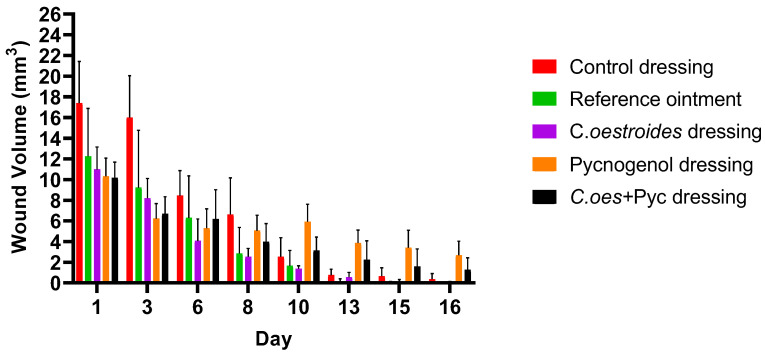
Wound volume measurements throughout the experiment. Significant differences were observed as follows: *C. oestroides* dressing and reference ointment showed significant differences compared to the other interventions (*p* < 0.001); Pycnogenol™ dressing showed significant worsening compared to the control and the *C. oestroides* treatments (*p* < 0.001).

**Figure 10:**
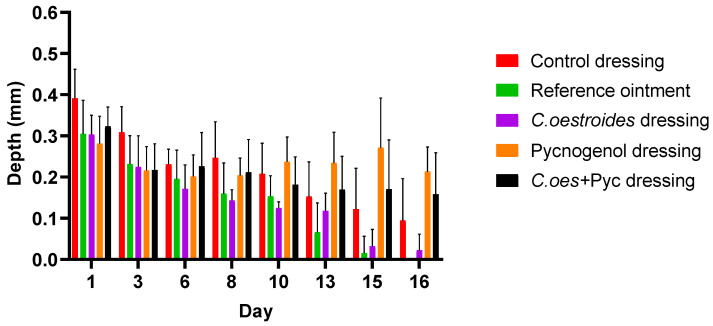
Wound depth measurements throughout the experiment. Significant differences were observed as follows: *C. oestroides* dressing and reference ointment showed a significant difference compared to the other interventions (*p* < 0.001); Pycnogenol™ dressing showed significant worsening compared to the control and the *C. oestroides* treatments (*p* < 0.001).

Significant differences were observed in the measurement of the surface area (Figure 8), volume (Figure 9), and depth (Figure 10) of the wounds among the diverse groups. By the 13th day, the reference ointment treatment showed a statistically significant difference compared to both the control dressing and the treatments with dressings containing Pycnogenol™ (*p* < 0.01). The analysis for the topical treatment with *C. oestroides* in dressing form on the 13th day was marginally non-significant (*p* = 0.067), but by the 15th day, the differences for this intervention became statistically significant (*p* < 0.02). It is noted that the two treatments only with *C. oestroides*, either in ointment or in dressing form, did not show statistically significant differences regarding wound size. Finally, the animals treated with dressings containing Pycnogenol™ showed a statistically significant worsening compared to the control and both *C. oestroides* treatments from the 6th day of the experiment, which continued until the 16th day (*p* < 0.001).

Comparable results were observed regarding the volume (Figure 7) of the wound and its depth (Figure 8) as the experiment ended. Again, the two *C. oestroides* treatments, whether in ointment or in dressing form, showed similar results and their results were significantly better than the other interventions (*p* < 0.001), while, at the same time, animals that received dressings containing Pycnogenol™ experienced significant worsening in comparison to both the control and the *C. oestroides*—only treatments (*p* < 0.001).

### 2.7. Photodocumentation

The results from the photodocumentation indicate that, by the 15th day, the group treated with the ointment containing *C. oestroides* experienced complete healing of all wounds, a phenomenon also observed for the group treated with a dressing containing only *C. oestroides* by the next (16th) day, as, on the 15th day, one wound appeared not to have fully healed.

**Figure 11:**
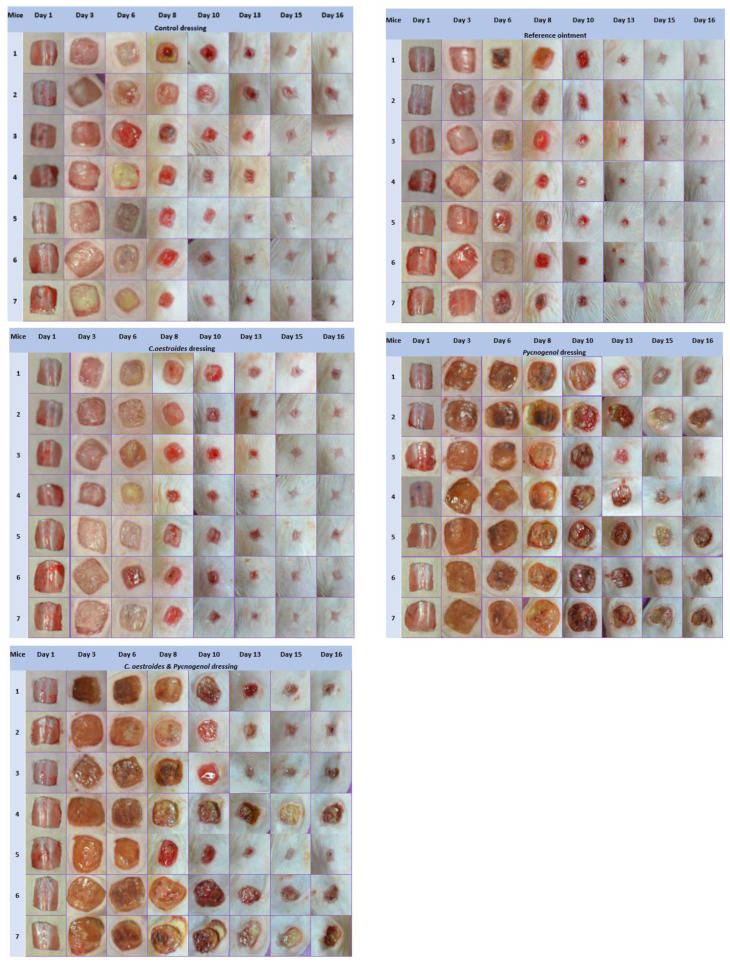
Photodocumentation throughout the experiment (days 1, 3, 6, 8, 10, 13, 15, and 16) display an evaluation of wound healing in the five treatment groups: control dressing; reference ointment; *C. oestroides* dressing; Pycnogenol™ dressing; *C. oestroides* + Pycnogenol dressing.

Conversely, in both groups treated with dressings containing Pycnogenol™, incomplete healing was observed in all cases. Lastly, the dressing that did not contain either of the two active agents showed healing, in most cases, by the 16th day (Figure 11).

### 2.8. Histopathological Findings

**Figure 12:**
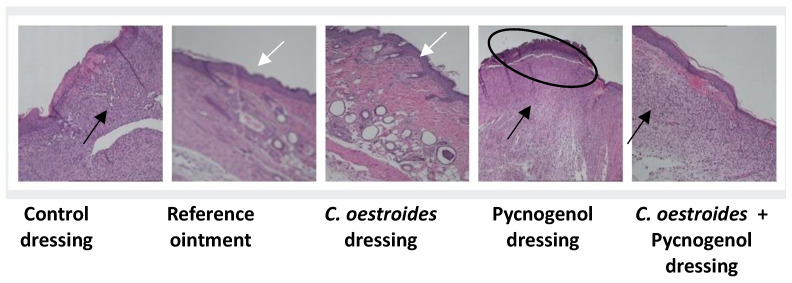
Skin sections at the end of the experimental procedure, stained with hematoxylin–eosin (100×). White arrows depict non-inflamed dermis; black arrows depict moderate to intense inflammatory elements; ellipses depict incomplete healing and parakeratosis.

In accordance with the histopathological observations of representative skin biopsies, the *C. oestroides*, with treatments scoring 6.5 and 7 (Table 1), showed complete healing with mild oedema and inflammation, and moderate hyperkeratosis in the post-traumatic tissue (Figure 12). 

In contrast, the experimental animals treated topically with dressings containing Pycnogenol™, both scoring 15 (Table 1), exhibited incomplete healing (ulceration) throughout the skin’s thickness, with intense inflammation, oedema, and hyperkeratosis. Significant parakeratosis was also observed (Figure 12).

Finally, in the case of the control, with a score of 11.25 (Table 1), the histological picture showed incomplete, partial thickness healing, with moderate inflammation, oedema, hyperkeratosis, and eventual parakeratosis (Figure 12).

### 2.9. Discussion

A new method to produce composite sodium alginate–maltodextrin dressings at low temperatures under vacuum was employed. This approach successfully produced stable and acceptable qualitatively dressings in a short period of time (Figure 1 and Figure 2). The method offers satisfactory results as a formulation technique for the *C. oestroides* extract, as it did not appear to compromise its efficacy, as seen in the provided figures (Figure 3, Figure 4, Figure 5, Figure 6, Figure 7, Figure 8, Figure 9 and Figure 10) and Table 1. The incorporation of active ingredients into dressings of is known to offer the benefits of a more precise dosing and extended release, thus enhancing the safety and effectiveness of topically administered active compounds [20,21]. Moreover, dressings offer a temporary protective physical barrier, soak up exudate from the wound, and maintain the moisture needed for optimal re-epithelialization [18,19,22].

Focusing on the results of the formulations containing *C. oestroides* olive oil extract, based on the observed outcomes, the extract has a positive impact on the healing process compared to the control. This confirms our laboratory’s previous observations regarding its excellent healing activity [11,12,13,14,15,16,17].

On the other hand, dressings containing Pycnogenol™, either alone or in combination with *C. oestroides* extract, exhibited undesired effects compared to the control and the formulations containing only *C. oestroides* (Figure 3, Figure 4, Figure 5, Figure 6, Figure 7, Figure 8, Figure 9 and Figure 10 and Table 1). This was the case even though the concentration used was the same as in previous experiments, which yielded significantly remarkable efficacy [7,8]. However, the presence of the *C. oestroides* extract seemed to mitigate these effects (Figure 6, Figure 7, Figure 8 and Figure 9). Under the current conditions, it was additionally noted that Pycnogenol™ resulted in a significant increase in body temperature and skin thickening in the experimental animals (Figure 3 and Figure 5). These findings corroborate the other observed results.

The histopathological evaluation further confirmed the above observations. It appears that the presence of Pycnogenol™ at this concentration in the dressings significantly delayed the healing process, while the examined samples (Figure 9 and Table 1) clearly showed both necrosis and parakeratosis, observations consistent with a local toxicity. On the other hand, in formulations containing *C. oestroides*, healing was complete and inflammation and oedema were mild, without necrosis or parakeratosis.

These phenomena could be attributed to the formulation of Pycnogenol™ into a dressing. In relation to a simple topical Pycnogenol™ gel preparation, the dressing could induce an enhancement of the concentration of Pycnogenol™ concentration due to a prolonged release at the wound site. Indeed, the higher concentration associated with prolonged contact at the site of the wound could induce toxic effects and therefore impair healing. Wounds have no stratum corneum and so there is no skin barrier to protect against excessive Pycnogenol™ penetration. This reveals a limitation of the current experiment, as different concentrations of Pycnogenol™ were not used to detect its potential beneficial action at a lower dose, as reported previously [8,9,10]. Unlike the extract from the isopod, Pycnogenol™ exhibits dose-dependent healing properties. This has been confirmed by both our laboratory [in-house unpublished data] and other researchers [23], and may be attributed to the fact that, at higher doses, Pycnogenol™ could act as a pro-oxidant or inhibit Toll-Like Receptors (TLR) [24], thereby hindering the healing process.

## 3. Conclusions

The method employed for producing the dressings seemed to yield satisfactory results concerning the formulation of the *C. oestroides* extract, indicating that the process did not compromise the extract’s healing properties. 

The ointment and dressing containing solely the *C. oestroides* extract demonstrated a significant healing effect compared to the control treatment and the dressings containing Pycnogenol™. This underscores the potential of the *C. oestroides* extract as a standalone treatment in promoting wound healing. On the contrary, dressings infused with Pycnogenol™, whether alone or in combination with the *C. oestroides* extract, were observed to exert a delaying effect on healing associated with local toxicity. However, this adverse impact appeared to be mitigated in the presence of the *C. oestroides* extract, confirming its significant healing properties. The toxic effect of Pycnogenol™ was also evidenced by an increase in body temperature, significant skin thickening, and a high area, volume, and depth of the wounds. The findings suggest a nuanced approach to the use of Pycnogenol™ in wound healing applications, underscoring the necessity for studies to delineate its optimal therapeutic dose-dependent window.

Overall, in the form of dressings, there is no doubt about the significant healing activity of *C. oestroides,* while further research is needed to determine the optimal dosage of Pycnogenol™. 

## 4. Materials and Methods

### 4.1. Reagents and Raw Materials

Sodium alginate, wool fat, and potassium hydroxide were purchased from Fagron Hellas SA (Trikala, Greece). Pycnogenol™ was generously provided by Horphag Research (Geneva, Switzerland). The olive oil was obtained from the local market (Lakonia, Greece). Specimens of *C. oestroides* were collected from infected seabreams fish farms (Chios, Greece). 

### 4.2. C. oestroides Olive Oil Extract Preparation

The extract was prepared by adding homogenized *C. oestroides* to olive oil at a concentration of 10% *w*/*w* and stirring for 24 h. After filtration, the extract was preserved at −20 °C.

### 4.3. Preparation of the Dressings

The dressings were produced following a two-step procedure. Initially, a gel containing appropriate ingredients for each intervention, as outlined in Table 2, was prepared. The gels were then evenly distributed into molds and weighed to ensure uniformity. Finally, the gels were vacuum-dried at 35 °C to a constant weight. The produced dressings were placed onto a nylon release liner and sealed in plastic bags, accompanied by silica gel sachets for moisture protection. The dressings were stored at room temperature. 

**Figure 13:**
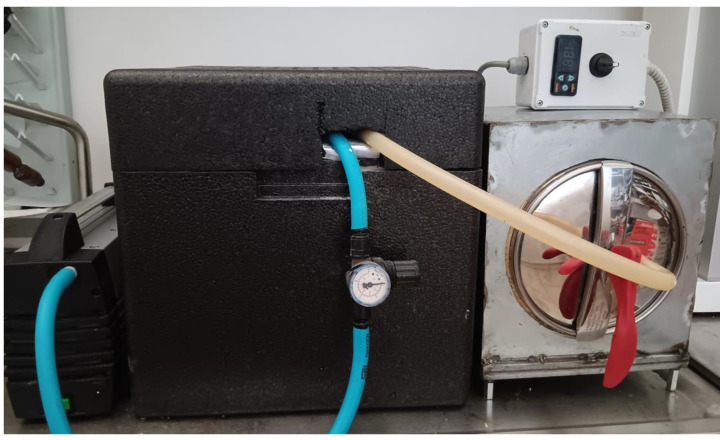
Custom-built apparatus for dressings production. On the right, there is a heated and thermostatically controlled vacuum chamber; in the middle, the vapors’ concentrating trap; and on the left, a vacuum pump.

The arrangement used to produce the dressings was a custom-built apparatus comprising three components. As evidenced in Figure 13, on the right, there was a heated and thermostatically controlled vacuum chamber, inside which a small fan was placed to ensure airflow within the chamber. In the middle, the arrangement for condensing vapors consisted of a vacuum container filled with silica gel, housed within a refrigeration unit maintained at 2–8 °C. Finally, the entire setup was connected to a vacuum pump (PM 23824-920, KNF Neuberger, Freiburg, Germany). The chamber was connected to the condensation container, and the condensation container was connected to the vacuum pump so that the air exiting the chamber would first pass through the condensation container before reaching the vacuum pump. Thus, the intermediate section of the device served as a trap for condensing vapors.

The doses of the active ingredients were set to 5% for Pycnogenol and 10% for the extract of *C. oestroides*. Both doses were selected based on previous relevant published studies that certified their efficacy at these concentrations [7,11,12,13,14,15,16,17].

### 4.4. Scanning Electron Microscopy (SEM)

All dressings were heightened after production. A PhenomWorld desktop scanning electron microscope (Thermo Fisher Scientific, Waltham, MA, USA) with a tungsten filament (10 kV) and charge reduction sample holder was used for the morphological characterization of the dressings and the samples were examined without sputter coating.

### 4.5. Sterility Control 

Upon completion of the production process, each batch underwent microbiological assessment to ensure a total bacterial count of zero, a total yeast count of zero, and the absence of pathogens (TMC, TYMC), in accordance with the methods established in the current European Pharmacopoeia.

### 4.6. In Vivo Study Design and Animals

All procedures were conducted in compliance with the guidelines set forth by the European Communities Council Directive (Directive 2010/63/EU of 22 September 2010). All study protocols adhered to the ARRIVE criteria [25]. 

Thirty-five male hairless mice, type SKH-hr2, aged 3–9 months, were utilized for this study. All mice originated from the breeding stock of the Department of Pharmacy Small Animal Laboratory (EL 25 BIO-BR 06). Experimental models in mice are very common for wound healing studies [26,27]. The number of animals was selected based on previously published similar studies. The temperature and humidity of the animal room were maintained at 24 ± 1 °C and 40 ± 10%, respectively, with the room illuminated under a 12 h cycle of light and dark. The mice had unlimited access to solid pellets (Nuevo SA, N. Artaki, Greece) and fresh water. 

The experimental procedure received approval from the National Peripheral Veterinary Authority Animal Ethics Committee (Protocol Number: 1113340-22/12/21). The animals were subjected to one week of acclimatization prior to the commencement of the experiment. The mice were stratified by age before being randomly divided into five groups of seven (n = 7) each, in accordance with the ARRIVE guidelines [26]. The age and weight were approximately the same across all groups. The study evaluated five different treatments, which included the following:

1. Dressings composed only of excipients, serving as a control, without therapeutic agents.
2. An ointment containing *Ceratothoa oestroides* extract, used as a positive control.
3. Dressings loaded with *Ceratothoa oestroides* extract.
4. Dressings loaded with Pycnogenol™
5. Dressings incorporating a combination of both *Ceratothoa oestroides* and Pycnogenol™ extracts.

The utilization of *C. oestroides* ointment as a reference ointment was based on our accumulated experience regarding the excellent healing properties of the formulation [11,12,13,14,15,16,17].

### 4.7. Wound Infliction

Initially, the animals were anaesthetized with an intraperitoneal injection of a combined solution of ketamine 100 mg/Kg (Narketan 10, 100 mg/mL Vetoquinol SA, Lure–Cedex, France) and xylazine 7 mg/Kg (Xylapan 20 mg/mL, France, France). Subsequently, a 1 cm^2^ piece of skin was surgically removed from the dorsal region of each mouse. After skin excision, the wounds were cleaned with normal saline solution and covered with fixed dressings (Fixomull, Beiersdorf AG, Hamburg, Germany).

### 4.8. Wound Maintenance

The wounds were cleansed with saline solution daily, and necrotic tissue and exudates were removed when deemed necessary. The different dressings used for each animal treatment were changed every 24 h. Specifically, for their application, they were initially cut into dimensions of 2 cm × 2 cm and then fixed at the site of inflammation with an adhesive dressing (Fixomull^®^, Beiersdorf) measuring 2 cm × 8 cm, which was wrapped around the body of the animal. To facilitate easy removal of the dressings before their removal, they were pre-moistened with saline solution.

### 4.9. Weight and Temperature Measurements

Monitoring of animal welfare was conducted through weight and temperature measurements at the beginning and the end of the experimentation. Weight measurements were performed using an electronic scale (KERN EHA 3000-0, KERN & SOHN GmbH, Balingen, Germany) and temperature measurements by a contactless infrared thermometer (NC150, Microlife, Windaus, Switzerland). Temperature measurements were conducted in triplicate.

### 4.10. Photodocumentation

Wounds were photographed after wound induction and every other day until the end of the experiments using a Nikon D5100 digital camera (Nikon, Tokyo, Japan) equipped with an AF-S Micro Nikkor 60 mm f/2.8 G ED lens (Nikon, Tokyo, Japan), which was fixed at a distance of 20 cm perpendicular to the subject. Pictures were also acquired using an Antera 3D camera (Miravex, Dublin, Ireland). Antera 3D uses an optical method combined with a complex algorithm to capture images in three dimensions. Its software, version 2.11.5, was used to evaluate wound area (mm^2^) and volume [28]. 

### 4.11. Evaluation of Transepidermal Water Loss (TEWL)

Transepidermal water loss (TWL or TEWL) is defined as the continuous evaporation of water that diffuses from the lower to the upper strata of the skin and subsequently passes into the environment as vapor. This measurement quantifies the function of the skin barrier. Measuring TEWL can be useful in identifying skin damage as TEWL rates increase with the severity of the damage. The evaluation of TEWL was performed using the Tewameter^®^ TM 240 (Courage-Khazaka, Cologne, Germany). This device features an electrochemical detector that indirectly determines water loss by recording temperature and relative humidity with two pairs of sensors inside a hollow plastic cylinder that is placed vertically on the skin. The measurements are expressed through a microprocessor as water loss relative to time and surface area (g/h/m^2^) [29]. 

### 4.12. Evaluation of Hydration 

The hydration measured here pertains to the water content of the keratin layer and not that of the entire skin. In normally hydrated skin, the moisture of the keratin layer is in the range of 10–16%. A decrease in moisture to less than 10% is accompanied by dryness, roughness, and brittleness. Conversely, an increase in water content to more than 16% causes overhydration, resulting in the loss of the compact structure of the keratin layer. The evaluation of hydration was conducted using the Corneometer^®^ CM 820 (Courage-Khazaka, Germany), which measures on a scale from 0 (no water content) to 120 (high water content), while the measurement units were established in the literature as “Corneometer units”.

The measurement is based on the capacitance measurement of a dielectric medium. During the measurement, the change in the dielectric constant due to hydration of the skin surface changes the capacitance of a precision capacitor. The penetration depth of the electric field of dispersion is proven to be exceedingly small, so only the moisture at the skin surface is measured. The duration of the measurement is noticeably short (1 s), thus preventing the formation of occlusion that would affect the outcome [30].

### 4.13. Evaluation of Skin Thickness

Skin thickness was measured using a digital caliper (Powerfix Prof Milomex Ltd., Bedfordshire, UK). Specifically, the thickness of the skin fold at the trauma area was determined. The device’s reading was presented in millimeters (mm).

### 4.14. Collection of Skin Samples—Histopathological Evaluation

At the end of the experiment (16th day), the animals were sacrificed, and biopsies of the skin area from their dorsal wound site were obtained. Pieces of the skin were preserved in formalin for histopathological evaluation.

The histopathological evaluation of the skin of the mice was conducted at the Pathology Laboratory of the Naval Hospital of Athens. The tissue samples were fixed in 10% formalin solution and then embedded in paraffin-forming paraffin blocks. Continuous sections were obtained and processed with hematoxylin and eosin (H&E) staining. Sections were examined under 100× magnification to assess inflammation, oedema, hyperkeratosis, wound thickness, and the presence of ulceration, necrosis, and parakeratosis, based on criteria outlined in Table 3.

### 4.15. Data Analysis 

All analyses and graphical representations were conducted using GraphPad Prism 8.4.2 (GraphPad Software, Inc., San Diego, CA, USA). A normality test of the data was conducted to decide whether to use parametric or non-parametric methods of analysis. For this purpose, the Kolmogorov–Smirnov and Shapiro–Wilk criteria were used. These criteria test whether the population distribution from which the random sample was drawn follows a specific probability distribution (e.g., normal). In all cases, it was found that the data followed a normal distribution (*p* > 0.05), allowing for the application of parametric methods of analysis.

To determine any statistically significant differences between treatments, analysis of variance (ANOVA) was applied. ANOVA is used to assess the statistical significance of differences in the means of more than two intervention samples. The results of ANOVA were evaluated by observing the significance value and applying the post-hoc Least Significant Difference (LSD) criterion. The threshold for significance in all tests was *p* ≤ 0.05.

The Student’s *t*-test for paired samples tests whether the population means between pairs of observations differ significantly from each other. In this case, we refer to the values of the same quantitative variable observed on the first and last day of the experiment.

## Figures and Tables

**Table 1:** Histopathological assessment results.

Specimens’ 16th Day	Ιinflammation	Oedema	Hyperkeratosis	Wound Depth	Ulceration	Necrosis	Parakeratosis	Score
Control dressing	2.25	2.5	2	3	1	0	0.5	11.25
Reference ointment	1	1	2	3	0	0	0	7
*C. oestroides* dressing	1	1	2	2.5	0	0	0	6.5
Pycnogenol™ dressing	3	3	3	3	1	1	1	15
*C. oestroides* and Pycnogenol™ dressing	3	3	3	3	1	1	1	15

Scoring criteria for histopathological evaluation in Section 4.14.

**Table 2:** Gel formulations from which dressings were prepared. All quantities are listed in grams.

Ingredients	Control (in Grams)	Pycnogenol™ and *C. oestroides* (in Grams)	*C. oestroides* (in Grams)	Pycnogenol™ (in Grams)
Pycnogenol™	-	1.2	-	1.2
Tapioca maltodextrin (Zorbit)	1	1	1	1
Sodium Alginate	4	4	4	4
Olive oil	2.4	-	-	2.4
*Ceratothoa oestroides* Olive oil extract	-	2.4	2.4	-
Wool fat (Lanolin)	4.5	4.5	4.5	4,5
Glycerin	10	10	10	10
Distilled water	78.1	76.9	78.1	76.9
Potassium hydroxide 50% Solution	.qs ad pH 6	.qs ad pH 6	.qs ad pH 6	.qs ad pH 6

**Table 3:** Scoring criteria for histopathological evaluation.

Scoring Criteria for Histopathological Evaluation				
Inflammation	0 (absence)	1 (mild)	2 (moderate)	3 (heavy)
Oedema	0 (absence)	1 (mild)	2 (moderate)	3 (heavy)
Hyperkeratosis	0 (absence)	1 (mild)	2 (moderate)	3 (heavy)
Wound thickness	0 (absence)	1 (superficial)	2 (moderate)	3 (total)
Ulceration	0 (absence)	1 (presence)		
Necrosis	0 (absence)	1 (presence)		
Parakeratosis	0 (absence)	1 (presence)		

## Data Availability

The data presented in this study are openly available in article.
